# Rewriting the Metabolic Blueprint: Advances in Pathway Diversification in Microorganisms

**DOI:** 10.3389/fmicb.2018.00155

**Published:** 2018-02-12

**Authors:** Gazi Sakir Hossain, Saravanan Prabhu Nadarajan, Lei Zhang, Tee-Kheang Ng, Jee Loon Foo, Hua Ling, Won Jae Choi, Matthew Wook Chang

**Affiliations:** ^1^Department of Biochemistry, Yong Loo Lin School of Medicine, National University of Singapore, Singapore, Singapore; ^2^NUS Synthetic Biology for Clinical and Technological Innovation (SynCTI), Life Sciences Institute, National University of Singapore, Singapore, Singapore; ^3^Agency for Science, Technology and Research (A^∗^STAR), Institute of Chemical and Engineering Sciences, Singapore, Singapore

**Keywords:** metabolic engineering, synthetic biology, pathway engineering, protein engineering, biochemical production

## Abstract

Living organisms have evolved over millions of years to fine tune their metabolism to create efficient pathways for producing metabolites necessary for their survival. Advancement in the field of synthetic biology has enabled the exploitation of these metabolic pathways for the production of desired compounds by creating microbial cell factories through metabolic engineering, thus providing sustainable routes to obtain value-added chemicals. Following the past success in metabolic engineering, there is increasing interest in diversifying natural metabolic pathways to construct non-natural biosynthesis routes, thereby creating possibilities for producing novel valuable compounds that are non-natural or without elucidated biosynthesis pathways. Thus, the range of chemicals that can be produced by biological systems can be expanded to meet the demands of industries for compounds such as plastic precursors and new antibiotics, most of which can only be obtained through chemical synthesis currently. Herein, we review and discuss novel strategies that have been developed to rewrite natural metabolic blueprints in a bid to broaden the chemical repertoire achievable in microorganisms. This review aims to provide insights on recent approaches taken to open new avenues for achieving biochemical production that are beyond currently available inventions.

## Introduction

**FIGURE 1:**
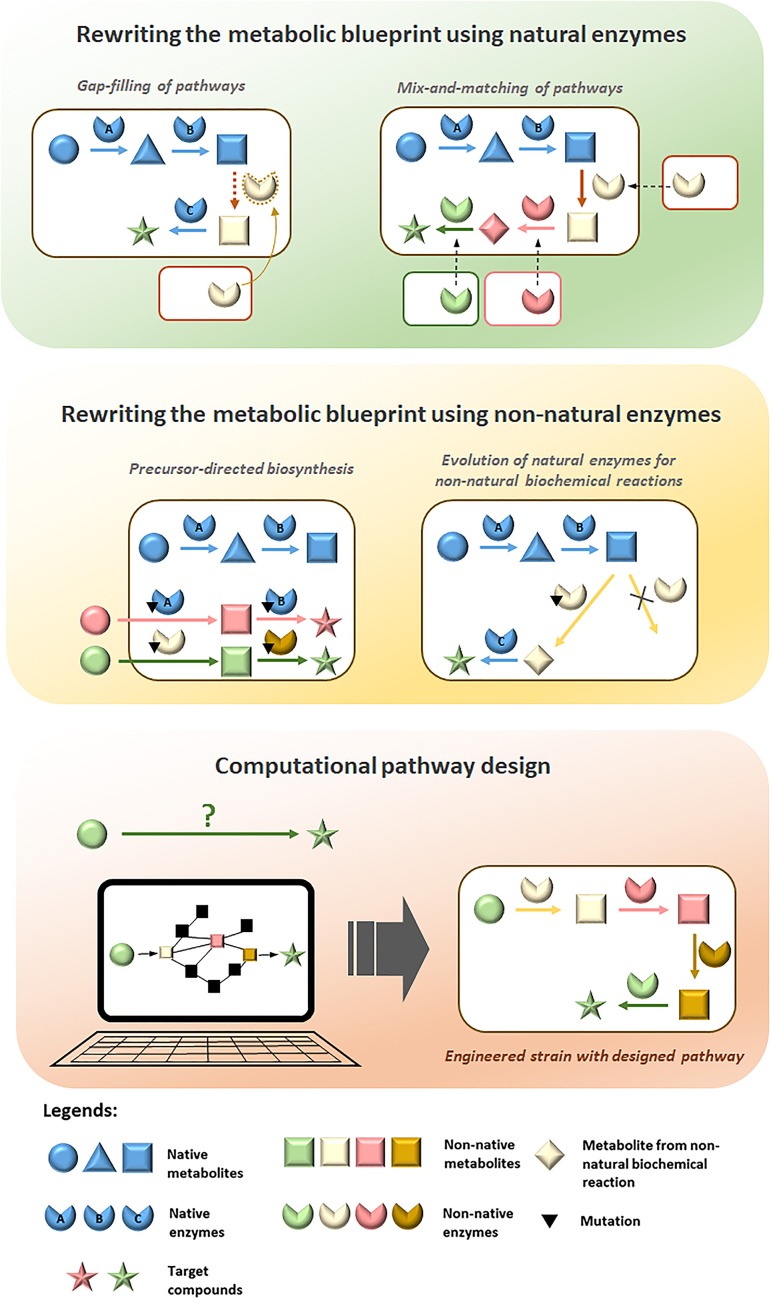
Overview of strategies employed for diversification of metabolic pathways to produce value-added compounds in microorganisms.

**FIGURE 2:**
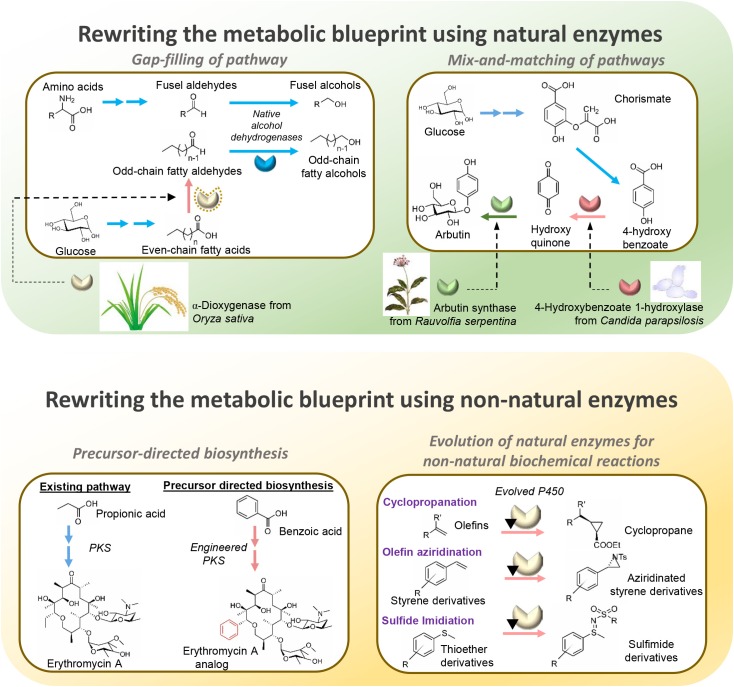
Illustration of distinct examples for each diversification approach.

Nature’s strength and beauty come from its diversity in biochemical systems, which not only generate but also degrade essential and non-essential chemical substances in living single cells or multicellular organisms through different biochemical reactions (i.e., metabolic pathways) that collectively form cellular metabolism. Consequently, a diverse range of biochemicals are present in nature. Many of these biochemicals are secondary metabolites to the native organisms but are of high biotechnological value to industries (Oksman-Caldentey and Inze, 2004; Harvey, 2008; Dhakal et al., 2017). Characterization of these secondary metabolites and exploration of the metabolic networks involved can potentially enable sustainable production of valuable and useful chemicals (Oksman-Caldentey and Inze, 2004). Although metabolic engineering has enabled the bioproduction of many valuable chemicals and has realized the aim of industrial-scale bio-based manufacturing of important compounds (e.g., 1,3-propanediol and artemisinin) (Zhu and Jackson, 2015), the range of compounds that can be generated are generally limited to those that occur naturally in living systems and with known biosynthesis pathways. Furthermore, the array of value-added chemicals required by industries is highly varied and many of these compounds can only be chemically synthesized as they are non-natural, and hence beyond the biosynthesis capabilities of existing biological systems. For example, different metabolic architectures have been identified from various organisms to produce carboxylic acids, which are central compounds in cellular metabolism (Lidén, 2017), thereby presenting promising sources for industrially important building blocks to manufacture bio-commodities, such as bioplastics. However, silicon-based materials can only be produced chemically because of the absence of organosilicon molecules in living organisms (Apeloig et al., 2001). Likewise, important secondary metabolites, such as polyketides and alkaloids, isolated from organisms (e.g., plants and marine microorganisms) can provide alternative therapeutics against health threats such as multidrug resistance bacteria and intractable cancers (Dhakal et al., 2016). Yet, while more potent analogs of these natural products may be discovered and obtained chemically (DeChristopher et al., 2012), novel biosynthesis routes to these superior non-natural drugs are elusive. Thus, it is imperative to diversify natural metabolic pathways to conceive novel ones that are capable of producing any desirable chemical through biological means. Herein, we will review novel strategies for rewriting natural metabolic blueprints and designing of biosynthetic pathways with computational tools (**Figures 1**, **2** and **Table 1**). We aim to provide current insights and future perspectives on how progress in the state-of-the-art approaches in metabolic pathway diversification for the production of novel value-added compounds will eventually facilitate the development of efficient designer microorganisms that can potentially meet most of the chemical needs of modern civilization.

**Table 1:** Examples of notable novel valuable compounds produced in microbial cell factories through diversification of metabolic pathways and computational tools developed for pathway design.

Products	Details of strategy	Reference
**Rewriting metabolic blueprint with natural enzyme**		

**Gap-filling of pathways**		
Nargenicin A1	Novel analog of nargenicin A1 was produced by expression of hydroxylase PikC from *Streptomyces venezuelae*.	Dhakal et al., 2016
Odd-chain fatty alcohol	Plant-derived α-dioxygenase enabled odd-chain length fatty aldehyde production from even chain fatty acid, further reduction led to formation of odd-chain fatty alcohol.	Jin et al., 2016
Long chain dicarboxylic acid	*Pseudomonas*-derived Baeyer–Villiger monooxygenase enzyme enabled production of dicarboxylic acid.	Song et al., 2014
Salvianic acid A	Metabolic engineering of *Escherichia coli* with D-lactate dehydrogenase achieved salvianic acid A production.	Yao et al., 2013
2-Pyrrolidone	Metabolic engineering of *E. coli* with 2-pyrrolidone synthase from *Streptomyces azureus* enabled 2-pyrrolidone production.	Zhang et al., 2016
Biofuels	Metabolic engineering of *B. coagulans* with citramalate synthase from *Methanococcus jannaschii* enabled synthesis of 2-ketoacid precursors.	Atsumi and Liao, 2008
D-Lactic acid	Synthesis of optically pure D-lactic acid was achieved in *E. coli* by expression of engineered glycerol dehydrogenase evolved for D-lactate dehydrogenase activity.	Wang et al., 2011
**Mix-and-matching of pathways**		
Arbutin	The skin-lightening agent was biosynthesized in *E. coli* by co-expressing 4-hydroxybenzoate 1-hydroxylase from *Candida parapsilosis* and arbutin synthase from *Rauvolfia serpentina.*	Shen et al., 2017
Polyketide analogs	Matching the D-desosamine and L-mycarose deoxysugar pathways with the alternative D-mycaminose and D-olivose pathways to produce new erythromycin analogs through the *E. coli* heterologous system.	Jiang et al., 2013
Opiates and related molecules	Production of opiate in yeast was achieved through the combination of new enzyme discovery, enzyme modification, and metabolic pathway optimization. Mixing and matching of 44 enzymes from bacteria, yeast, plants, and mammals were required.	Galanie et al., 2015
Phenylpropanoid derivatives	Seven biosynthetic genes from plants and bacteria were mixed and matched in *E. coli* to produce phenylpropanoid acids, stilbenoids and curcuminoids.	Wang et al., 2015
4-Ketozeinoxanthin	4-Ketozeinoxanthin was produced in *E. coli* by mixing and matching of carotenoid ketolase gene from marine bacteria, lycopene biosynthesis genes from soil bacterium *Pantoea ananatis* and lycopene β-cyclase, lycopene 𝜀-cyclase, β-carotenoid hydroxylase from liverwort *Marchantia polymorpha*.	Maoka et al., 2014
Unnatural multi-methyl-branched butyl esters (MBEs)	MBEs were produced by engineered *E. coli* where mycocerosic polyketide synthases (PKSs) were mixed and matched from *Mycobacterium tuberculosis*. Expression of those enzymes enabled the biosynthesis of MBEs by utilizing various lengths of fatty acid with linear and branched chain alcohols.	Menendez-Bravo et al., 2014

**Rewriting metabolic blueprint with non-natural enzyme**		

**Precursor-directed biosynthesis**		
Polyketides	Substrate promiscuity of crotonyl-CoA carboxylase/reductase (CCR) homologs enabled the production of unnatural polyketide derivatives.	Wilson and Moore, 2012
Andrimid analog	Precursor-directed evolved non-ribosomal peptide synthetase (NRPS) produced three derivatives of the antibacterial compound, andrimid, in the native producer, *Pantoea agglomerans*. The compounds were analogs of the natural product with diverse functionality.	Evans et al., 2011
Fluorinated polyketide	Directed evolution-generated polyketide synthase enabled utilization of fluorinated building blocks to produce novel fluorinated scaffold of polyketide.	Walker et al., 2013
Tetracycline analogs	Novel tetracycline analogs with different scaffolds were produced using a new set of tailoring enzymes.	Wang et al., 2012
Calcium dependent antibiotics	Engineering the adenylation domain of NRPS enabled novel NRP production using non-natural amino acid as precursor.	Thirlway et al., 2012
Nitro-substituted polyketide aureothin	Rational design and directed evolution of aureothin modular polyketide synthase enabled diverse polyketide production.	Sugimoto et al., 2014
Unnatural alkaloid scaffold	Novel polyketide-alkaloid hybrid molecules were produced using precursor-directed and structure-based design.	Morita et al., 2011
Erythromycin A analog	Tailoring the enzymes of erythromycin gene cluster enabled benzoate to be used as a precursor leading to the formation of novel erythromycin derivative.	Zhang et al., 2010;Jiang and Pfeifer, 2013
Clorobiocin analog	3,4-Dihydroxybenzoic acid was produced *de novo* as precursor for the biosynthesis pathway of the antibiotic clorobiocin to generate a potent analog.	Alt et al., 2011
Alkyne labeled polyketide	Heterologous expression of a terminal alkyne-forming operon with PKS/NPRS genes in *E. coli* enabled alkyne-labeled polyketide production from hexenoic acid.	Zhu et al., 2015
**Evolution of natural enzymes for non-natural biochemical reactions**		
2,4-DHB	Novel products were produced by evolving enzymes in an aspartate-using pathway for previously unreported activities to create an analogous malate-utilizing pathway.	Walther et al., 2017
Cyclopropanes and olefin metathesis	Engineered cytochrome P450 enabled selective carbene transfers from diazoesters to olefins for cyclopropanation.	Coelho et al., 2013
Sulfimides	Evolved cytochrome P450 catalyzed imidation of sulfides to form sulfimides.	Farwell et al., 2014
Aziridines	Engineered cytochrome P450 achieved intermolecular aziridination using tosyl azide and styrenes as substrates.	Farwell et al., 2015
Organoboranes	Directed evolution of cytochrome c enabled synthesis of organoboranes.	Kan et al., 2017

**Computational pathway design**		

fastGapFill	fastGapFill reconstructs metabolic networks by identifying enzyme candidates from universal reaction databases to gap-fill missing pathways.	Thiele et al., 2014
BoostGAPFILL	BoostGAPFILL predicts missing biochemical reactions in metabolic networks based on metabolites present, including non-native ones.	Oyetunde et al., 2017
RetroPath	RetroPath is a computation tool that automates metabolic pathway design for given sets of specifications, including precursors and target chemicals.	Carbonell et al., 2014
Pinocembrin	A five-enzyme pathway was designed using RetroPath to successfully produce pinocembrin in *E. coli* from malonyl-CoA.	Feher et al., 2014

## Strategies to Diversify Metabolic Pathway for Synthesis of Value-Added Compounds

In order to produce novel compounds biologically, it is essential to rewire the native metabolic pathways in production hosts to form new ones that will lead to the desired compounds. Non-natural biosynthesis routes need to be designed, implemented and optimized through metabolic engineering for efficient production of the target compounds. In this section, we will review efforts in production of value-added compounds accomplished through rewriting of metabolic network with natural and non-natural enzymes, as well as computational design of non-natural metabolic pathways.

### Rewriting the Metabolic Blueprint Using Natural Enzymes

Enzymes vary widely in the reactions they catalyze and in their substrate specificities. Homologs are present across different species thus there is a vast number of enzyme candidates that can be selected from nature for construction of novel pathways. By introducing non-native enzymes from different organisms into a production host, metabolic rewiring can be achieved to expand possibilities for biosynthesis of novel compounds. Two different approaches have been utilized to rewrite metabolic networks using natural enzymes: gap-filling and mix-and-matching of pathways. Here, we will review the employment of these strategies for diversifying metabolic pathways to produce value-added compounds.

#### Gap-Filling of Pathways

Native metabolic pathways in organisms are connected and insulated from one another to varying degrees (Hartwell et al., 1999). By gap-filling pathways with heterologous enzymes, shunts can be built between the pathways to create new and diverse biosynthesis routes toward desired metabolites (Shin et al., 2013; Lee and Kim, 2015). Essentially, by introducing a suitable non-native enzyme, a metabolite from a native pathway can be converted by the heterologous enzyme to a non-native intermediate to serve as a substrate of a previously disconnected native pathway, thus creating a novel biosynthesis pathway for producing target compounds. For example, the biosynthesis of odd-chain fatty alcohols, which is of industrial value, is made possible by the heterologous expression of α-dioxygenase from *Oryza sativa* in *Saccharomyces cerevisiae* (Jin et al., 2016). The α-dioxygenase can convert endogenous even-chain fatty acids to odd-chain fatty aldehydes which are subsequently reduced by native alcohol dehydrogenases to produce odd-chain fatty alcohols (Jin et al., 2016). Notably, although the biosynthesis pathway of salvianic acid A in the plant *Salvia miltiorrhiza* was unclear, the therapeutic antioxidant was remarkably produced in *Escherichia coli* by rerouting endogenously produced 4-hydroxyphenylpyruvate with D-lactate dehydrogenase derived from *Lactobacillus pentosus* to 4-hydroxyphenyllactate for conversion by a native hydroxylase complex to salvianic acid A (Yao et al., 2015). Similarly, novel nargenicin A1 derivatives were synthesized in *Nocardia* by introducing a hydroxylase (PikC) derived from the pikromycin gene cluster of *Streptomyces venezuelae* (Dhakal et al., 2016). The gap-filling strategy was also employed for the efficient synthesis of optically pure D-lactic acid in high titers by utilizing a glycerol dehydrogenase engineered for D-lactate dehydrogenase activity to gap-fill the pyruvate metabolism in *Bacillus coagulans* (Wang et al., 2011). Likewise, to produce biofuels such as propanol and butanol in high yield, a new pathway was engineered in *E. coli* for the production of 2-ketoacid precursors from pyruvate and acetyl-CoA by utilizing citramalate synthase from *Methanococcus jannaschii* (Atsumi and Liao, 2008). Other notable value-added chemicals produced by the gap-filling strategy, such as long-chain dicarboxylic acid (Song et al., 2014) and 2-pyrrolidone (Zhang et al., 2016), are summarized in **Table 1**. These examples illustrate that although gap-filling is a simple approach, relying on expression of a heterologous enzyme to diversify metabolic pathways, it demonstrates great potential in expanding the range of bio-based chemicals that can be produced. However, a major disadvantage of the gap-filling strategy is that it is applicable only when an intermediate metabolite can be found that links two native pathways to produce the target compound, thus limiting the possibilities of attainable pathway diversification. To allow greater diversification of biosynthetic pathways, rewiring of metabolic networks needs to be more extensive using other strategies such as mix-and-matching of pathways.

#### Mix-and-Matching of Pathways

The mix-and-match approach combinatorically expresses multiple genes or clusters from numerous organisms in a production host to produce metabolites of interest (**Figure 1**). Given the large number of annotated enzymes with known functions, artificial biosynthesis routes can therefore be built rationally by mix-and-matching suitable non-native enzymes to diversify native pathways toward target compounds. This strategy is particularly useful for reconstruction of pathways that are not fully elucidated and is exemplified by the production of the skin lightening agent arbutin in *E. coli*. Although the natural pathway for biosynthesizing arbutin is not fully understood, it was successfully produced in *E. coli* by diversifying the chorismic acid metabolic pathway with 4-hydroxybenzoate 1-hydroxylase from *Candida parapsilosis* to produce hydroquinone, which serves as the substrate for arbutin synthase from *Rauvolfia serpentina* to produce arbutin (Shen et al., 2017). The mix-and-match approach is also an effective strategy for producing variants of natural products, such as polyketides, carotenoids, phenyl propanoids and alkaloids, which involve gene clusters for biosynthesis. Genes in diverse sets of homologous gene clusters from different organisms can be viewed as “modules” for combinatorial assembly to produce novel derivatives. Using this strategy, novel pathways were constructed to enable biosynthesis of compounds with therapeutic and commercial value (**Table 1**). Taken together, the mix-and-match approach has demonstrated immense potential for diversifying metabolic pathways using combinations of natural enzymes for the production of novel value-added compounds. Nevertheless, mix-and-matching of pathways is often marred by low productivity due to substrate specificity constraints since the new metabolites are not natural substrates of the enzymes in the pathways assembled. Thus, the full potential of the mix-and-match strategy could only be realized by involving non-natural enzymes that have been evolved to suit the needs of the novel pathways.

### Rewriting the Metabolic Blueprint with Non-natural Enzyme

Diversification of natural metabolic pathways to create novel biosynthesis routes will inevitably form intermediate metabolites that are beyond the substrate range which the natural enzymes involved can perform efficiently. Although nature has provided us with a gamut of enzymes to choose from, generation of non-natural enzymes is often essential for successful construction of efficient novel pathways to extensively rewrite metabolic blueprints (Foo et al., 2012). As most natural enzymes exhibit degrees of substrate promiscuity to analogs of their natural substrates (Gupta, 2016), promiscuous properties of natural enzymes can be exploited through protein engineering approaches to evolve their activities toward non-natural substrates. In addition to employment in gap-filling and mix-and-matching of pathways, non-natural enzymes have important applications for precursor-directed biosynthesis and catalysis of non-natural biochemical reactions, which are powerful strategies that we will review in this section for bio-based production of novel value-added chemicals.

#### Precursor-Directed Biosynthesis

Precursor-directed biosynthesis involves using evolved enzymes with altered substrate specificity to incorporate structurally diverse analogs of natural substrates into novel metabolic pathways in order to produce non-natural biochemicals. Commonly, substrate analogs are provided *ex vivo* to production hosts with non-natural enzymes with relaxed substrate specificity to form novel intermediates. While it is possible to rely on substrate promiscuity of the natural enzymes in the native pathways to convert the intermediates to the desired natural product derivatives, engineered enzymes are frequently required for the pathways to be efficient. For example, acyltransferase, the “gatekeeping” enzyme to polyketide synthesis, has been engineered to accept analogs of natural acyl-CoA precursors to initiate production of novel polyketide derivatives (Dunn and Khosla, 2013). Inclusion of enzymes modified by protein engineering in the downstream pathway can greatly facilitate the conversion of the resulting non-natural intermediates to a wide range of polyketide derivatives with potential therapeutic properties, as exemplified by the work of Lee et al. (2011) to create derivatives of the antibiotic erythromycin. Similar approaches were applied to enable biosynthesis of a wide range of novel derivatives of polyketides, non-ribosomal peptides (NRPs) and phenyl propanoids (**Table 1**). *De novo* precursor-directed biosynthesis can be achieved by co-expressing a pathway for generating substrate analog *in vivo* instead of *ex vivo* supplementation. This was demonstrated by Alt et al. for producing a novel derivative of the antibiotic clorobiocin by employing a 3,4-dihydroxybenzoic acid-producing pathway to provide the non-natural precursor to the clorobiocin biosynthesis pathway, resulting in the production of a potent DNA gyrase inhibitor (Alt et al., 2011).

With these examples, precursor-directed biosynthesis has proven to be an effective approach for generating a wide array of novel compounds rapidly with non-natural precursors. However, these precursors are often expensive and could only be synthesized chemically. Therefore, generating enzymes that can catalyze non-natural reactions are vital for progressing diversification of metabolic pathways.

#### Evolution of Natural Enzymes for Non-natural Biochemical Reactions

Metabolic engineering has advanced by leaps and bounds over the past decades facilitating the biosynthesis of many valuable compounds for pharmaceutical and industrial applications. However, chemical synthesis still has an edge in terms of the versatility of chemical structures that can be generated because biological synthesis is limited by the number of natural enzymes available (Wallace and Balskus, 2014). To circumvent this constraint, efforts have been directed toward creating enzymes that can perform reactions that have not been possible biologically for constructing novel metabolic pathways to produce compounds without known natural biosynthesis routes. For example, the versatile chemical, 2,4-dihydroxybutyric acid (2,4-DHB), was produced by exploiting a natural metabolic pathway involving aspartate to utilize malate, a structurally similar analog, as precursor (Walther et al., 2017). By engineering the enzymes in the natural pathway, novel enzymes with previously unreported activities in nature, namely malate semialdehyde reductase, malate kinase, and malate semialdehyde dehydrogenase, were generated to create an artificial 2,4-DHB-producing pathway. This illustrates the importance of non-natural enzymes for diversifying metabolic pathways. In recent years, numerous evolution and engineering strategies were applied to mechanistically diverse the superfamilies of biocatalysts to perform novel reactions (Gerlt and Babbitt, 2009). For instance, cytochrome P450s have been evolved to perform olefin aziridination (Farwell et al., 2015), carbene transfer to olefin (Coelho et al., 2013), and imidation of sulfides (Farwell et al., 2014), reactions which are unknown in the biological world. The promising trend of growing number of novel enzymes catalyzing non-natural biochemical reactions suggest the possibility of constructing fully artificial pathways to biosynthesize any compound that can be accomplished chemically.

### Computational Pathway Design

As computing power grew exponentially in the past decade, computational tools became increasingly attractive as toolkits for metabolic engineering. While these tools initially were employed mainly to optimize metabolic pathways for achieving production of target compound with high yield (Copeland et al., 2012), they have been extended to facilitate designing of novel pathways. For example, to complement a rapid increase in the number of sequenced genomes, computational methods were created for data mining to identify and annotate genes (Blanco and Abril, 2009). However, it has reached a stage where the data generated exceeds the rate that they can be sifted and organized (Khosla, 2015). Automated annotation of these “big data” simply based on sequence homology has been unreliable or unsuccessful half of the time (Gerlt, 2016). To understand relationships between proteins in the databases, the “big data” generated could greatly benefit from tools developed by the Enzyme Function Initiative, such as EFI-EST and EFI-GNT (Gerlt, 2017), which use multidisciplinary approaches to accurately assign enzyme functions (Gerlt, 2016). Proper functional assignment is vital for identifying novel enzyme candidates from diverse superfamilies that can be deployed to construct non-natural biosynthesis routes. However, for diversification of metabolic pathways, creation of tools to predict and identify plausible routes are imperative to accelerate the design process for integrating heterologous genes to produce novel compounds in defined hosts. Therefore, computational tools such as BoostGAPFILL (Oyetunde et al., 2017) and fastGapFill (Thiele et al., 2014) have been developed to enable identification of candidate enzymes from a universal biochemical reaction database to fill network gaps. To enable the design of biologically feasible pathways to produce any target compound of interest, the retrosynthesis approach, which is a well-established method used in organic chemistry for identifying suitable precursors and synthesis routes, was combined with biological knowledge to create tools such as RetroPath (Carbonell et al., 2014) to automate the pathway design process. Several possible pathways consisting of various enzymes that can lead precursors to the target compound can be proposed and ranked based on selected criteria. Subsequently, RetroPath was successfully applied to design a pathway for biosynthesizing the antioxidant flavonoid pinocembrin, resulting in a five-enzyme pathway starting from malonyl-CoA as precursor (Feher et al., 2014). These promising computational tools and results suggest that computational pathway design will greatly facilitate the design of novel pathways and continued efforts in improving these computational suites will enable expansion of the chemical repertoire that can be produced biologically.

## Conclusion and Future Perspectives

Metabolic engineering has progressed tremendously over decades with the aim to efficiently produce biochemicals from renewable resources to serve industrial needs. Indeed, there are many examples of engineered microbes that are able to produce industrially relevant levels of biochemical for commercialization (Zhu and Jackson, 2015). However, metabolic engineering is still largely limited to production of chemicals that exist naturally in biological systems. Therefore, the range of chemicals that metabolic engineering can generate is unable to meet that required by industries. Consequently, there is still heavy reliance on chemical synthesis from fossil resources for precursors needed by industries (Wallace and Balskus, 2014). Hence, diversification of metabolic pathways is crucial to achieve biosynthesis of non-natural compounds to rival the capabilities of chemical synthesis. Herein, we have reviewed several strategies that have been developed in the attempt to broaden the spectrum of compounds that can be created biologically. While the results have been promising, much still needs to be done before biological systems can be utilized to produce any target compound of choice.

Being a well-established field on its own, protein engineering is an area that can greatly benefit and complement efforts in metabolic pathway diversification (Foo et al., 2012). By increasing the number of available biocatalysts to catalyze non-natural reactions, protein engineering tools act as a driving force for designing new biosynthesis routes to create novel compounds. Further, these tailor-made enzymes catalyzing novel non-natural reactions uncover new arena to establish novel metabolic pathways. One remarkable example of the capability of protein engineering is the evolution of a natural enzyme to create carbon-silicon bonds, which are important moieties in many commercial products but do not exist in nature, essentially opening up possibilities of creating silicon-based biosynthetic pathways (Kan et al., 2016). Likewise, evolved *Rhodothermus marinus* cytochrome *c* have been employed to enable carbon–boron bond formation in *E. coli* to produce organoboranes, a class of compounds that are not found in biological systems but with applications in chemotherapeutics (Kan et al., 2017). With rapid advances in genome sequencing techniques, more enzymes will be identified to serve as candidates for engineering non-natural biochemical reactions. This may be complemented by the recent boom in microbiome studies, where metagenomics techniques have been developed and applied to sequence genomes of many microorganisms that were previously uncultivable. Computation-guided genome mining can thus be applied to discover novel natural products and their biosynthetic pathways (Medema and Fischbach, 2015).

Ultimately, biosynthesis pathways are a series of synchronized chemical reactions performed in biological chassis. Therefore, to create diverse non-natural metabolic pathways, it is imperative to incorporate chemical knowledge and techniques, particularly retrosynthesis, to complement our understanding of biological systems when designing biochemical routes required to produce novel target compounds. As discussed earlier, combining computational tools with retrosynthesis has already achieved some success in designing non-natural pathways for the biosynthesis of novel value-added compounds (Feher et al., 2014). By further developing user-friendly computational tools and databases that include all known natural and non-natural enzymes, automated platforms that integrate current strategies and knowledge pertinent to pathway diversification could be established to significantly accelerate designing and implementation of novel pathways.

Despite rapid advancement in the field of metabolic engineering and the potential tools available for interplaying with genetic material and metabolic networks of microbial workhorses, there are major bottlenecks that require resolution when forward engineering metabolic pathways. Alterations to metabolic pathways with heterologous or engineered enzymes in microbial hosts burden the microbial host and cause issues such as imbalance in metabolic pathways, poor growth, accumulation of toxic intermediates and other physiological stresses. Moreover, conventional model strains commonly used as production hosts often face issues under the harsh operating conditions of downstream industrial processes. These problems may be overcome by selecting suitable production hosts beyond the conventional ones, depending on the target compounds (Czajka et al., 2017). For example, the non-conventional oleaginous yeast *Yarrowia lipolytica* was exploited for production of fatty acid-derivatives (Zhu and Jackson, 2015). Furthermore, *Pseudomonas* strains possess metabolic, physiological and stress-tolerance characteristics that are favorable for metabolic engineering (Nikel et al., 2014) and *Pseudomonas putida* was demonstrated to be an ideal host for production of *para*-hydroxybenzoic acid due to its exceptional tolerance for aromatic compounds (Yu et al., 2016). In addition to strategic selection of production host, computation-guided pathway designing approaches, such as genome scale modeling and novel machine learning methods, have proven to be alternative solutions to solve metabolic imbalance issues by efficient strain engineering strategies (Wu G. et al., 2016; Wu S.G. et al., 2016). Concurrently, the lack of natural enzyme reaction cascade in engineered pathways brings about inefficient substrate conversion. To address this issue, artificial enzyme channels have been constructed to organize enzymes for efficient production of target compounds. This strategy has been reviewed extensively in literature (Proschel et al., 2015). These examples demonstrate that extensive efforts are ongoing for strain development to implement synthetic pathways efficiently, which is immensely beneficial for pathway diversification.

In conclusion, much progress in diversification of metabolic pathways has been made and the future for production of non-natural chemicals using biological systems is promising. There are fundamental concerns regarding the compatibility of non-natural pathways and products with living systems, such as substrate availability, toxicity of substrates and products, availability and intracellular balance of cofactors, and influx/efflux of substrates and products, that will impede the yield and productivity of the biosynthesis routes. Nevertheless, there are many tools available for strain engineering, such as targeted multi-site CRISPR/Cas9-based gene insertion or deletion (Jakociunas et al., 2015), riboswitch-based self-directed evolution of phenotype (Pham et al., 2017) and Synthetic Chromosome Recombination and Modification by LoxP-mediated Evolution (SCRaMbLE) in Synthetic Yeast 2.0 (Dymond and Boeke, 2012), that may be utilized to optimize host strains for tolerance towards non-natural pathways and products. Impressively, synthetic biology recently enabled the creation of a semi-synthetic organism to possess two additional letters in its codons that form an unnatural base pair. The organism can thus store increased genetic information and potentially create enzymes with novel activities, e.g., by incorporating non-canonical amino acids, thereby facilitate diversification of metabolic pathways (Zhang et al., 2017). In future, intensive research in pathway diversification might enable development of highly efficient microbial strains that can potentially carry out desired chemical reactions to produce any target compounds and eventually serve as a sustainable source for supplying valuable biochemicals to meet industrial needs.

## Author Contributions

JLF, HL, WJC, and MWC conceptualized the content, figures, and table of the review. GSH, SPN, and LZ wrote the manuscript. JLF and HL revised the manuscript. T-KN and MWC proof corrected and reviewed the manuscript.

## Conflict of Interest Statement

The authors declare that the research was conducted in the absence of any commercial or financial relationships that could be construed as a potential conflict of interest.
